# On Complex Coacervate Core Micelles: Structure-Function Perspectives

**DOI:** 10.3390/polym12091953

**Published:** 2020-08-28

**Authors:** Jose Rodrigo Magana, Christian C. M. Sproncken, Ilja K. Voets

**Affiliations:** Laboratory of Self-Organizing Soft Matter, Department of Chemical Engineering and Chemistry and Institute for Complex Molecular Systems, Eindhoven University of Technology, P.O. Box 513, 5600 MB Eindhoven, The Netherlands; j.r.magana.rodriguez@tue.nl (J.R.M.); c.c.m.sproncken@tue.nl (C.C.M.S.)

**Keywords:** co-assembly, complex coacervate core micelle, interpolyelectrolyte complex, polyelectrolytes, polyion complex, polyplex, protein-polymer complex, structure-function relations

## Abstract

The co-assembly of ionic-neutral block copolymers with oppositely charged species produces nanometric colloidal complexes, known, among other names, as complex coacervates core micelles (C3Ms). C3Ms are of widespread interest in nanomedicine for controlled delivery and release, whilst research activity into other application areas, such as gelation, catalysis, nanoparticle synthesis, and sensing, is increasing. In this review, we discuss recent studies on the functional roles that C3Ms can fulfil in these and other fields, focusing on emerging structure–function relations and remaining knowledge gaps.

## 1. Introduction

The hierarchical assembly of biomacromolecules into superstructures plays a pivotal role in many biological functions, such as signal transduction, motility, cell growth, and differentiation. For example, the docking of proteins onto DNA is the primary cellular mechanism to regulate transcription. Enzymes are often activated to function upon supramolecular polymerization into dimers or tetramers. The chemical diversity of sequence-controlled biopolymers and their intricate interaction pathways lead to spatiotemporal variations in composition and abundance, which govern the creation and dissolution of a plethora of well-defined complexes to perform virtually all functions essential for life. Many strategies to develop adaptive materials are inspired by these concepts and exploit the controlled co-assembly of multiple, custom-tailored building blocks into mixed association colloids with mesoscopic dimensions. Modulation of the function of such materials is then attainable by fine-tuning of the chemical nature, arrangement, and interactions of and between the constituents. In this review, we discuss recent advances in the field of nanometric association colloids assembled from mixtures of oppositely charged polymers (and other compounds) due to electrostatic and other non-covalent interactions. We focus in particular on the functional aspects of this novel class of adaptive, polymeric materials and highlight how structure-function relations may serve as guidelines for their rational design and development. The interested reader is referred to excellent reviews on the fundamentals and theory of these polymeric association colloids, which are briefly discussed but not addressed in-depth herein [1,2,3,4,5,6,7].

## 2. Fundamentals

The co-assembly of oppositely charged polymers has attracted considerable attention, since it provides a robust and intuitive platform to prepare multi-responsive and multifunctional polymeric nanoparticles. These materials marry the responsivity and functionality of the different types of constituent (co)polymers within a single compartment of nanometric dimensions. Many of these polymeric association colloids form as a consequence of the electrostatic interactions between two oppositely charged, hydrophilic polyelectrolyte chains in water, which gives rise to an associative phase separation under specific conditions. Over time, two coexisting macroscopic liquid phases develop if, e.g., hydrophilic homopolymers are mixed, but the macroscopic phase separation can be restricted to the colloidal scale by attachment of a neutral water-soluble block to one or both polyelectrolytes. The tethered neutral block then prevents the coacervate from growing further, resulting in a (coacervate) core/shell micelle or vesicle, instead of a two-phase, liquid-liquid system with a dilute phase depleted of polymer and a denser coacervate phase enriched in both polyelectrolytes. The architecture of the copolymer(s) can be of a different nature. Whilst block copolymers are the most common, graft or random copolymers can also be utilized [8,9,10,11,12].

**Figure 1:**
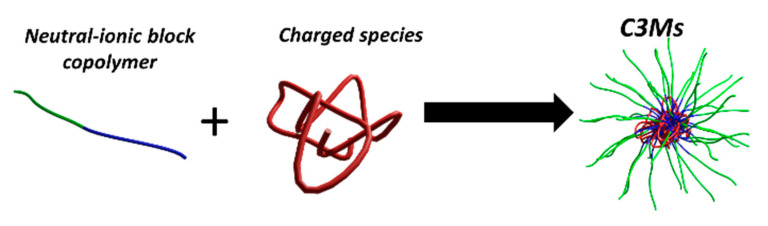
Schematic representation of the formation of complex coacervate core micelles (C3Ms) from a neutral-ionic block copolymer and an oppositely charged species in aqueous solution.

Since ionic-neutral copolymers co-assemble with a broad range of oppositely charged compounds, these have been exploited to encapsulate many different types of chemical species, including linear block (co)polymers, biopolymers, such as DNA [13,14], proteins [15], surfactants [16], metallic complexes [17,18], nanoparticles, and dendrimers [19]. The resultant core/shell hydrocolloids are referred to in the literature as complex coacervate core micelles (C3Ms), polyion complex (PIC) micelles, block ionomer complexes (BIC) and (micellar) interpolyelectrolyte complexes (IPEC), among others. Throughout this review, we will employ the term C3Ms for these particles (Figure 1), regardless of the physical state of the core, even though, strictly speaking, this term only applies to micelles with a core comprising a coacervate (i.e., liquid) phase. The complexation of two or more suitable ionic-neutral copolymers and/or terpolymers may also yield multi-compartment micelles, with a (partially) segregated core and/or corona [20]. Janus micelles (or vesicles) with a laterally phase segregated corona have been reported [21], as well as onion-type micelles, consisting of a hydrophobic core, a coacervate inner corona, and a neutral outer shell [22,23,24]. The (internal) structure and morphology of such complex colloids are determined by a subtle interplay of many factors, including, but not limited to, polymer composition, architecture, cohesive interactions, miscibility, and differential solvency. 

**Figure 2:**
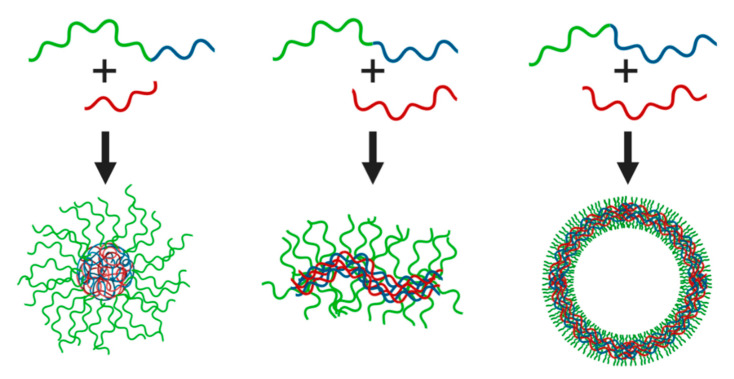
Schematic representation of the different assemblies formed by oppositely charged (block co)polymers. Relative block lengths can dictate the formation of spherical micelles (**left**), wormlike micelles (**middle**) or vesicles (**right**) with a complex coacervate core.

Interestingly, virtually all of these association colloids are stimuli-responsive, since the strength and length of the non-covalent bonds between the constituents are dependent on salt concentration, salt type, and pH in case of weak polyelectrolytes with a pH-dependent charge density. This is due to the electrostatic driving forces for complexation, which include salt-dependent enthalpic and entropic contributions. At low salt concentrations, counterion release and tight ion-pairing considerably decrease the free energy of the system upon micellization [25]. Auxiliary driving forces, such as hydrophobic forces, may have a major impact on the properties of C3Ms. For example, the mechanical properties and salt-stability of polyelectrolyte complexes comprising poly(styrenesulfonate) (PSS) and poly(4-vinylpyridine) (P4VP), quaternized with increasingly longer alkyl side chains, varied markedly upon tuning the cation hydrophobicity [26]. Longer alkyl chains provided stronger hydrophobic forces and thus higher salt stability. The incorporation of light- and temperature-responsive moieties enables further modulation of the interaction forces with external cues [27,28]. Moreover, the type and mixing fraction of chargeable monomers, the relative block lengths of the polyelectrolyte blocks, and various other compositional parameters can be adjusted to custom-tailor the phase behavior and structure-function relations. Analogous to amphiphilic (surfactant or polymeric) micelles, C3M shapes can be varied by choice of the block length ratios. Relatively long neutral soluble blocks yield spherical micelles, but shorter corona-forming blocks can result in wormlike micelles or vesicles (Figure 2). Additionally, a transition from spherical to elongated complexes can be induced by increased salt concentration, by compacting and swelling of the respective corona- and core-forming polymers [29]. This uniquely adaptive character is not only fundamentally attractive but also appealing for utilization of the particles as, e.g., nanocarriers or nanoreactors [30,31]. C3Ms find widespread interest in the nanomedicine community for controlled delivery and release, whilst research activity into other application areas, such as gelation, catalysis, and sensing, is increasing. Table 1 provides an overview of some polymers and oppositely charged species used for different applications. In this review, we discuss recent studies on the functional roles that C3Ms can fulfil in these and other fields, focusing on emerging structure-function relations and remaining knowledge gaps. 

## 3. Biotechnological Applications of C3Ms

In the last few decades, medicine has mainly relied on the use of small therapeutic drugs to control, reverse, and stop diseases. These small therapeutic drugs are not intrinsically efficient and selective. Regular and sustained administration is required in many cases to maintain their therapeutic action [32]. These hurdles may induce unwanted adverse effects. On the other hand, biologics, such as proteins and polynucleotides, are inherently selective, highly efficient, and perform specific functions that rely on triggered responses upon exposure to specific substrates present in vivo. Moreover, as they are similar in composition to the main components in the body, immunogenicity and toxicity are typically lower compared to small molecular drugs. For these reasons, biologics are gradually becoming the new standard in the medical field. This trend is clearly manifested in the drug market sales worldwide, since many of the most profitable therapeutics available today are biologics such as enzymes, antibodies, peptides, viruses, and nucleic acids [33,34,35]. Addressing the many challenges associated with the delivery and release of biologics, including those that are in clinical use, is thus of considerable fundamental and applied interest.

Biologics are difficult to deliver into the targeted tissue principally because of their low colloidal and structural stability, degradation in physiological media, and limited cell internalization. Most biologics benefit from a polymeric shell. For example, encapsulating biologics inside liposomes, capsules, viral capsids, and nanoparticles can remarkably improve their colloidal stability and protect them from in vivo degradation. In this context, C3Ms are advantageous, because the hydrophilic coacervate core allows the encapsulation of fragile, water-soluble substances. Polynucleotides, proteins, peptides, and ionic drugs can be buried and protected in the C3M core, without loss of structural and functional integrity, while this is typically unsuccessful or inefficient in classical micelles from amphiphiles.

The modular preparation of C3Ms is often advantageous since the physicochemical properties, such as size, surface charge, and shape, can be finely tuned by varying monomer type and the (block) length (ratio) of the copolymer. Which copolymers are best selected for the preparation of C3Ms custom-tailored for a specific purpose depends on the relation between their composition and the structural and functional properties of the resultant micelles, including biocompatibility, blood circulation time, and biodegradability (Table 1A). For C3Ms to be suitable as nanocarriers for biologics, the micelles must display both high colloidal stability during transit and triggered dissociation upon environmental changes in the targeted tissue. To meet these often-conflicting demands, C3Ms can be programmed by the incorporation of appropriate chemical functionalities to respond to specific intracellular signals, such as the local ATP concentration, acidic pH values (endosomes) and reducing conditions (cytosol). The C3M shell not only serves to protect the cargo during transport without eliciting an immunogenic response, but may also be modified chemically for targeting purposes to enhance the accumulation and promote internalization at the desired locations [7].

### 3.1. Polynucleotide-Based C3Ms

The number of clinical studies on nucleic acid-based therapies is steadily increasing owing to the versatility and high efficacy of gene therapies with plasmid DNA (pDNA), small interference RNA (siRNA), antisense RNA (mRNA) [33]. The potency of these high potential treatments can be further improved if the two major bottlenecks of limited physiological stability and inefficient targeting can be tackled successfully. For example, free polynucleotides are digested by nucleases within minutes in the bloodstream. Moreover, the electrostatic repulsive interactions between the charged polynucleotides and the cell membrane result in poor cellular uptake. These challenges motivated the development of suitable delivery systems to protect the polynucleotides at physiological conditions against degradation, so that the material remains intact upon arrival at the active site.

Sequestering the genetic material into virus capsids is the most widely used approach in gene therapy. Virus capsids are outstanding delivery vehicles, but they are expensive to produce and difficult to manipulate. An appealing alternative strategy entails the complexation of the negatively charged genetic material with cationic polymers to form so-called polyplexes. Using cationic-neutral block copolymers instead of cationic homopolymers generates C3Ms, which increases biocompatibility, survival in the bloodstream, and transfection efficiency [36].

**Figure 3:**
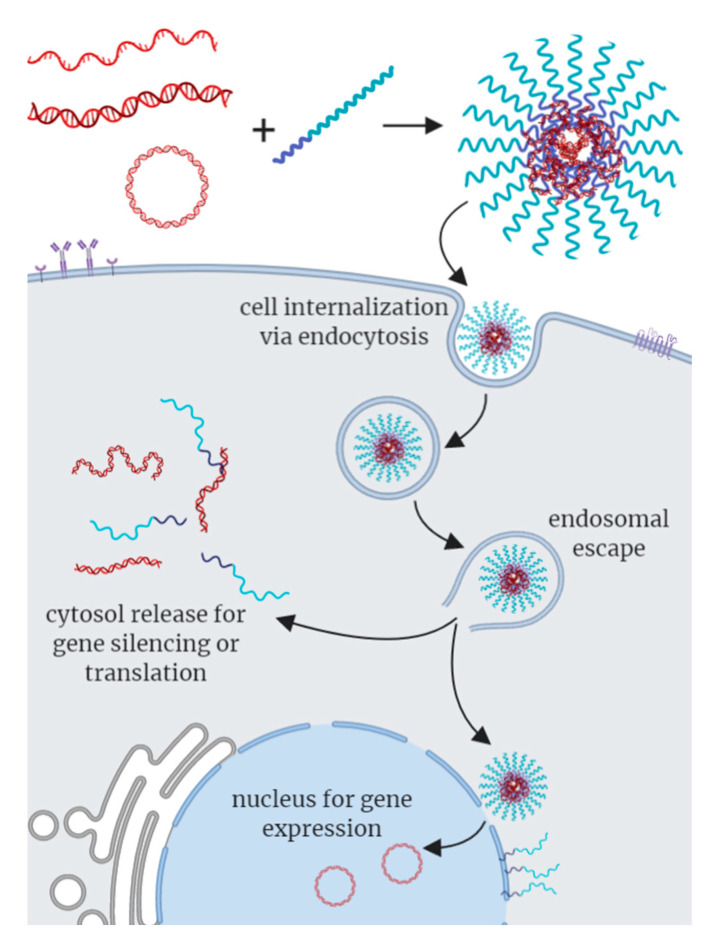
Schematic representation of uptake of polynucleotide-based C3Ms by cells. Single-stranded DNA, double-stranded DNA and/or plasmid DNA may form polynucleotide-based C3Ms upon complexation with a cationic-neutral block copolymer. C3Ms are internalized via endocytosis. Disruption of the endosomal membrane leads to escape of the cargo, after which the genetic material can be released into the cytosol, or the C3M may interact at the nuclear membrane to release the DNA into the nucleus.

To fully exploit their application potential, polynucleotide-based C3Ms must have superb colloidal stability and maintain structural integrity under physiological conditions, promote cellular uptake, facilitate endosomal escape, and ultimately deliver the genetic cargo into the cytosol or nucleus (Figure 3). The neutral hydrophilic block in C3Ms, often poly(ethylene glycol) (PEG), stabilizes the micelles and reduces the cytotoxicity of the polyelectrolyte. In addition, the chain end of the neutral block can be functionalized with ligands, such as lactose and peptides, to target specific organs [37,38,39]. C3Ms are typically prepared under charge stoichiometric conditions and, therefore, neutral, which effectively increases DNA cellular uptake. It is also possible to prepare C3Ms under non-stoichiometric conditions to render C3Ms with a positive surface charge or to include ligands in the shell, to further facilitate cellular uptake and endosomal escape. Despite these attractive features, DNA- and oligonucleotide-based C3Ms are inefficient compared to viral vectors. Boosting the performance of C3Ms to (out) compete with viral vectors has, thus, become a central objective in nanomedicine. In the following, we highlight key findings and recent advances towards this aim.

A critical steppingstone towards systemic gene therapies based on C3Ms is the preparation of micelles with high stability in the bloodstream. Neutral-cationic block copolymers generally yield relatively stable C3Ms when mixed with polynucleotides; however, they may be dissociated if there are competitive charged species in solution such as endogenous RNA, glycosaminoglycans and negatively charged polysaccharides [40]. Additionally, shear forces inside the blood vessels can also break down C3Ms [41]. Colloidal stability is increased by intracellularly reversible crosslinking [42,43,44], the introduction of hydrophobic moieties in the block copolymer [45,46,47,48], covalent grafting of RNA to a polymer backbone [49], or oligomerization of the encapsulated polynucleotide chains to increase their molecular weight and thereby the cohesion of the C3M [50,51]. A particularly important design challenge for polynucleotide-based delivery systems is the protection of the genetic material against nuclease digestion. The PEG shell of C3Ms delays, but does not entirely block, the nuclease-driven degradation of the packaged genetic material. Ultimately, after several hours, the DNA is cleaved [52]. Incorporation of a hydrophobic intermediate block in the copolymer enhances stability towards nuclease digestion as it creates a barrier around the genetic material, which retards diffusion of the nucleases into the micellar core. To this end, thermo-responsive blocks, such as poly(2-n-propyl-2-oxazoline) (PnPrOx) and poly(*N*-isopropyl acrylamide) (PNIPAM), which are soluble at room temperature and insoluble at body temperature, have been introduced between the neutral and cationic blocks [47,53].

The nature of the cationic polyelectrolyte is a critical design parameter, since it impacts both transfection efficiency and toxicity. Unfortunately, polyelectrolyte optimization is often a double-edged sword: when transfection efficiency improves concomitantly toxicity rises, and vice versa, less toxic compounds typically display lower transfection efficiency. For example, 25 kDa poly(ethylene imine) (PEI) produces the highest transfection rate among all charged blocks used in polyplexes, but is highly cytotoxic [54]. PEGylation of the PEI improves cell viability at the expense of lower cellular uptake, endosomal escape, and consequently, transfection efficiency. High molecular weight polymers show enhanced DNA binding, cellular uptake, and transfection efficiency, while low molecular weight polymers are less cytotoxic and can efficiently unpack DNA after transfection [55,56,57]. Aiming for the best of both worlds, Reineke et al. developed short, linear, neutral-cationic copolymers by placing biocompatible carbohydrates between oligo-amines (PEI), resulting in so-called poly(glycoamidoamine) (PGAA) [58,59,60]. Remarkably, the polyplex transfection rates are high due to the PEI chain, while the protecting carbohydrate block and the low molecular weight reduce the immune response and cytotoxicity. The preparation of polyplexes in excess PGAAs renders a positive surface charge, which increases the interaction with negatively charged proteins on the cell wall and promotes endocytosis. Systematic variations in the carbohydrate type and amount of charges, as well as their sequence in PGAAs, showed that transfection efficiency is influenced significantly by several different factors, including the increase in charge upon exposure to endosomal pH, endosomal escape, and also the binding strength of the polymer to the oligonucleotide [61].

After cell incorporation via endocytosis, the polynucleotide-based C3Ms must escape from the endosome before they are trafficked to late endosomes, lysosomes, or other organelles. Endosomal escape is assumed to be caused by the destabilization of the endosomal membrane; a mechanism referred to as the “proton sponge effect” [62]. Some empirical evidence supporting this hypothesis suggests that upon exposure to endosomal pH (ca. 5.5), the charge density of the polycations increases, which facilitates the intercalation of the polymer into the anionic endosomal membrane, accelerating its disruption and endosomal escape of the polynucleotides. However, recent super-resolution optical microscopy studies reveal that oligonucleotide-based C3Ms remain intact after the endosomal escape, which suggests that it is the positively charged surface of the C3Ms at endosomal pH that destabilizes the membrane [63]. Some of the most efficient polycations include, PEI [64,65], poly(histidine) [66], dendrimers [19], and poly(aspartamide) [67,68]. Additional hydrophobic groups in the block copolymer, such as cholesterol, also facilitate endosomal escape as these promote interaction with the lipidic bilayer [13]. It is worth noting that the neutral block in a cationic-neutral copolymer may obstruct the electrostatic interaction of the polycation with the endosomal membrane, causing a decrease in the efficiency of endosomal escape, even for polycations, which are very efficient in membrane disruption. An effective means to tackle this hurdle is the inclusion of intracellularly cleavable linkers between the neutral and charged blocks of the block copolymers, such as sulphide- or boron-based bonds, which are sensitive to pH or carbohydrate molecules [69,70].

Control over C3M size and morphology is also essential for C3M-mediated gene delivery because these properties are directly related to the efficiency of cellular uptake and clearance from the body [71,72,73]. Under stoichiometric conditions, C3Ms are typically spherical core-shell structures [12]. However, since oligonucleotides are strong and stiff Pes, they may adopt different morphologies, depending on the salt conditions and ratio between the neutral and cationic block. Tirrel et al. compared the structure of C3Ms composed of poly(ethylene glycol)-*block*-poly(l-lysine) PEG-*b*-PLL and single-stranded DNA (ssDNA) with C3Ms comprising PEG-*b*-PLL and double-stranded DNA (dsDNA) [74,75]. As observed previously for C3Ms comprising exclusively linear diblock copolymers and homopolymers [29], the length of the block copolymer determined the size of DNA-containing C3Ms. Complexation with ssDNA produced spherical micelles, while cylindrical micelles formed when dsDNA was incorporated instead. The charge density and rigidity of ssDNA vs. dsDNA defined the C3M morphology to optimize the packing of the polynucleotides in the micellar core. Small-angle X-ray scattering profiles and cryo-transmission electron microscopy of dsDNA polyplexes displayed a sharp Bragg peak located at 2.7 nm, corresponding to the *d*-spacing reported for the interhelix distance in genomic DNA toroids [76]. L.Shen et al. prepared polyplexes using polymerization-induced electrostatic self-assembly (PIESA) between siRNA or DNA and a growing chain of 3-acrylamidopropyl trimethylammonium chloride (APTAC) from a PEG block. While DNA-based C3M presented mainly spherical morphologies, the rigidity of the short siRNA [19–21 bp] played a role in directing the assembly resulting in the formation of C3Ms with unusual morphologies [77]. Upon a systematic increase in the degree of polymerization (DP) of PAPTAC, lamellae, tubes, and spheres were created for low (<40), intermediate and high (>70) DPs, respectively.

Interestingly, pDNA, which is more flexible than small oligonucleotides, can also adopt toroidal and rod-shaped structures, when complexed with neutral-cationic block copolymers, to allow the pDNA to be folded in a relaxed conformation [29,47,78,79,80,81]. Rod-like C3Ms with a modular rod length were prepared from PEG-*b*-PLL and pDNA. A 3.5-fold decrease in the length of the cationic block of the diblock copolymer from 20 to 70 decreased the length of the rod-like micelles 10-fold from hundreds of nanometres to 70 nm [73]. Fine-tuning of the length of the PLL block of PEG-*b*-PLL is crucial, as it affects cellular uptake and resistance against nuclease degradation. Shorter PLL blocks reduce cellular uptake as this rendered the PEG shell more crowded [81]. Cellular uptake was improved for longer PLL blocks but also reduced nuclease stability, as this generated globular micelles with a low-density PEG shell [82]. Interestingly, the highest cell internalization and transfection efficiency were observed for a rod length of ca. 200 nm, which is consistent with the upper size limit of the clathrin-dependent endocytic vesicles.

Whereas oligonucleotides do not need to translocate into the cell nucleus for gene expression, this is essential for DNA. New developments in optical microscopy recently enabled, for the first time, imaging of the fate of internalized C3Ms with nanometric resolution [63,83,84]. This shed light on various aspects of nucleus internalization, although much remains to be elucidated. pDNA-based C3Ms must remain intact after endosomal escape, because free pDNA is rapidly digested in the cytosol. Two-color direct stochastic optical reconstruction super-resolution microscopy revealed that pDNA-based C3Ms concentrated in the perinuclear region after cell incubation for approximately 12 h [84]. Passive translocation of DNA-based C3Ms can be achieved in dividing cells, where the nuclear membrane dissociates during cell mitosis. It appears—although it is still debated how—that C3Ms and/or pDNA may also penetrate the nucleus via the nuclear pore complex, which contains small pores of 5–10 nm in diameter [85,86]. Whether C3Ms remain intact after nucleus penetration is yet to be established as is the intranuclear transfection mechanism. Interestingly, cell-free studies have shown that transcription can also undergo within C3Ms, which suggests that C3Ms need not dissociate in the nucleus for the genetic code to be read [82]. Consequently, having a proper pDNA conformation and distribution in the C3Ms may enhance the efficiency of the vector substantially [52,80,81]. The potency of this concept is perhaps best illustrated by the superior transfection efficacy of micelleplexes compared to the golden standard jetPEI (25 kDa linear PEI) and polyplexes. Whereas 70% of HEK293T cells were successfully transfected, when subjected to micelleplexes with pDNA encoding for green fluorescence protein (GFP), only 40% and 10% of HEK293T were transfected when instead jetPEI and conventional polyplexes were utilized. This dramatic improvement in transfection efficiency is presumably due to the preservation of the native conformation of pDNA within micelleplexes, which are essentially aggregates of pDNA and positively charged micelles [87]. The pDNA configuration in micelleplexes appears analogous to the DNA compaction by histones. This allows greater accessibility to the payload thereby promoting protein expression compared to conventional polyplexes, which tightly condense pDNA, and may significantly distort its structure. 

### 3.2. Encapsulation of Proteins in C3Ms

Many treatments take advantage of the evolutionary honed specificity and efficiency of proteins. From a therapeutic perspective, enzymes render low cytotoxicity, high specificity, and efficiency, which reduces the risk to elicit an adverse immune response and cause side effects. Unfortunately, the chemical and structural stability of these biomacromolecules in a physiological environment is often insufficient; a complication that must be overcome to bring their potential as biologics to full fruition [35]. Non-native pH, (multivalent) salts, temperature, and proteases may (locally) disrupt folding or (partially) degrade the enzyme, compromising activity significantly. The encapsulation of proteins into nanocarriers to protect the biopolymers and preserve the native fold and activity under non-native conditions has, thus, become an active field of research and development. C3Ms are an appealing nanocarrier for these purposes since packaging within the core of such micelles improves the stability of the enzymes, and high protein loading by mass is attainable [88]. The associative phase separation between the protein and an ionic-neutral block copolymer can result in C3Ms with sequestered enzymes. The water-rich core provides the enzyme with a near-native environment to preserve its activity and structure. Similar to encapsulation within C3Ms of DNA, complexation with oppositely charged copolymers improves protein stability with respect to ionic strength, dilution, denaturation by urea, and proteases. Importantly, the rather high water content of the C3M core also allows for diffusion of relatively small compounds into and out of the C3M, so that the catalytic sites of the incorporated enzymes remains accessible for entry and exit of substrates and products. An overview of proteins and polymers used to prepare C3Ms is given in Table 1B.

The central objective of efforts to fine-tune the structure and properties of enzyme-loaded C3Ms to further improve their performance as nanocarriers of biologics is to strike a balance between high enzymatic activity and long-term (colloidal) stability on the one hand and triggered release on the other hand. Other design considerations include encapsulation efficiency and biocompatibility. The amphoteric nature and heterogeneous charge distribution of the cargo compromise the (long-term) stability and efficient loading of C3Ms with enzymes. Compared to a linear polyelectrolyte of the same mass, proteins carry far less solvent-accessible charges for complexation, and moreover, the protein surface typically displays charged amino acids with both the same and opposite sign as that of the ionic-neutral copolymer. Not all enzymes are amenable to encapsulation within C3Ms; in some cases, less well-defined structures without a core/shell architecture are formed. The number of proteins per C3M and their internal distribution are often unknown. Additionally, a stoichiometric charge ratio (i.e., when the concentration of chargeable monomers of the copolymer equals the net protein charge concentration) usually does not lead to complete charge neutralization, because the polymer is not flexible enough to compensate all accessible surface charges of the protein, consequently, requiring more polymer chains to neutralize the protein charges. It is also notable that reaching the equilibrium after C3M formation can take anywhere from seconds to up to several days, depending on the protein, polymer, and salt concentration used [89,90]. Clearly, the design rules for C3Ms formation between oppositely charged copolymers are not directly applicable to protein-containing C3Ms, as their assembly does not depend purely on electrostatics. Several routes may be taken to tackle these challenges and to design and characterize a protein-polymer complex tailored for specific needs. Here, we discuss some of the main strategies for producing protein-loaded C3Ms, highlighting their advantages, as well as limitations, and what steps are yet to be explored.

#### Strategies for Enhanced Protein Encapsulation and Stabilization

Some naturally occurring proteins associate directly with polyelectrolytes because of their high surface charge at acidic or basic pH values [15,89,90,91,92]; however, most proteins have a near-neutral surface charge at physiological conditions. How to effectively incorporate proteins into C3Ms has thus become a central research question in the field. Much is still to be unravelled on the matter. For example, the correlation between protein structure and loading capacity is not well understood yet. Interestingly, the amount of proteins encapsulated in a coacervate appears to depend on more factors than solely on protein size and net charge. Encapsulation of proteins using cationic copolymers is promoted under acidic conditions, e.g., at pH values below the isoelectric point (pI) of the protein, so that the net surface charge density is sufficiently high. Vice versa, encapsulation using anionic copolymers is more efficient at basic pH values above the pI. However, coacervation can also occur at pH values on the so-called ‘wrong side’ of the isoelectric point as a consequence of the high charge heterogeneity on the protein surface [93,94].

Due to the amphoteric nature of the protein surface, small variations in pH and salt concentration may be detrimental to C3Ms stability. In many cases, the protein charge may be too low to produce stable C3Ms. Systematic studies on multiple supercharged proteins showed that complex coacervation requires at least 30% excess of either basic or acidic amino acids [90]. The stability of C3Ms notably improves if proteins are incorporated into the C3Ms core together with a like-charged polyelectrolyte [95,96,97,98]. Interestingly, varying the ratio between the protein and homopolymer allows the control of the number of enzymes in the coacervate. This strategy was used, for example, for the electrostatic association of poly(oligo(ethylene glycol) methyl ether methacrylate)-*block*-poly(*N*-methyl-4-vinylpyridinium iodide) (POEGMA-*b*-PM4VP) with poly(acrylic acid) (PAA) and organophosphate hydrolase (OPH). The C3Ms were stable towards high temperatures, as the enzymatic activity of the complexes, compared to the native protein, was almost 2-fold higher after incubation at 37 °C for three days. Additionally, activity was still retained in the presence of organic solvents, such as ethanol or dimethyl methylphosphonate, since the aqueous fraction and charge-rich environment in the coacervate core stabilize the enzyme against organic solvent denaturation. Interestingly, the coacervates prepared with only OPH and POEGMA-*b*-PM4VP were less stable than those prepared with the ternary mixture [99]. 

**Figure 4:**
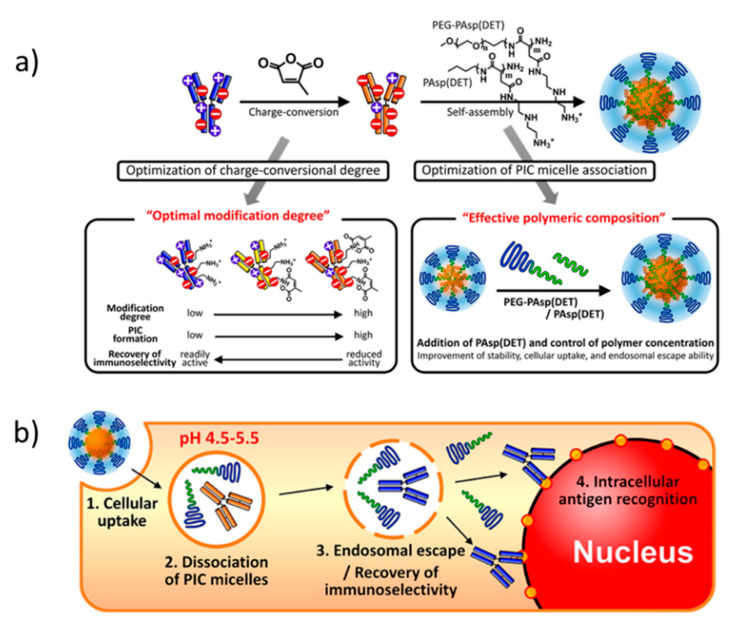
(**a**) Charge conversion of immunoglobulin with citraconic anhydride and subsequent C3M formation with poly(ethylene glycol) (PEG)-*b*-PAsp(DET) block copolymer and PAsp(DET) homopolymer. (**b**) schematic mechanism for C3M cell internalization, dissociation, and recovery of native immunoglobulin. Figure adapted from Kim et al. (doi:10.1021/acs.biomac.5b01335) as published by the American Chemical Society [102]. Further permission to this material should be directed to the ACS.

Supercharging of the enzyme surface is a powerful means to render proteins amenable for coacervation [89,90]. Modifying the surface accessible lysines with carboxylates or aspartic and glutamic acids with tertiary amines, to reverse their charge, yields high charge density enzymes. The chemical functionalization of the amino acids, however, might compromise enzymatic activity in certain cases, e.g., when the active site is modified. Protein engineering can deliver supercharged proteins with a custom-tailored charge distribution; however, this strategy is specific for recombinantly expressed proteins and does not apply to commercially or post-expressed proteins [89,100]. Reversible chemical reactions are an attractive route to overcome these limitations. For example, citraconic amides, formed by reacting citraconic anhydride with lysines, are fully reversible at pH = 5.5 [101,102]. C3Ms prepared by mixing poly(ethylene glycol)-*block*-poly[*N*-{*N*-(2aminoethyl)-2-aminoethyl} aspartamide] (PEG-*b*-PAsp(DET)) with citraconic anhydride-modified immunoglobulin achieved cell internalization and efficient cytosol delivery (Figure 4) [102]. Supercharging reduced the capacity of the immunoglobulin to bind the cell nucleus, while it promoted encapsulation into C3Ms, and thereby stabilized the antibody under physiological conditions. After cell internalization via endocytosis, the labile amide bonds were cleaved in the acidic endosomal environment, which restored the native and active state of the monoclonal antibody. Subsequently, the native antibody was released into the cytosol. Interestingly, the addition of PAsp(DET) homopolymer during C3M formation improved stability, cellular uptake, and endosomal escape. The latter is in line with previous studies on cellular uptake and endosomal escape of polyplexes composed of cationic homopolymers and cationic-neutral diblock copolymers, which indicate a more favorable interaction of the homopolymers with the cellular and endosomal membranes, so that cell internalization and endosomal escape are enhanced.

Conjugation of charged (bio)polymers and oligomers to the termini of proteins, either during expression or post-purification, is another appealing route to supercharging to promote encapsulation of proteins in C3Ms. This strategy is widely applied to enhance protein loading into other nanocarriers, such as lipidic vesicles, nanoparticles, and amphiphilic micelles [103,104,105]. Likewise, in nature, proteins with charged (and intrinsically disordered) regions are more likely to associate with oppositely charged macromolecules [106]. Recently, Obermeyer et al. demonstrated the feasibility of this principle by recombinant expression and complexation of green fluorescent protein (GFP) equipped with non-native C-terminal short [Asp–Glu–Glu–Glu–Asp–Asp] repeating segments as an auxiliary charged tag to promote coacervation [107]. The main advantage of this method of supercharging, which we think has great potential, is that the tags barely perturb protein activity and structure. Another innovative strategy worthy of further exploration is based on the specific interaction between multivalent Ni^2+^ ions and short histidine segments, which are commonly included in recombinantly expressed proteins for Ni-NTA purification. For example, anionic polyelectrolytes comprising nitriloacetic acid groups complexed with Ni^2+^ may serve as a platform for the non-covalent attachment of affinity-purified hexahistidine tagged proteins [108]. This preloaded polyelectrolyte could be used as a constituent to prepare C3Ms with high protein-loading efficiency.

As discussed in the above, relying exclusively on electrostatic interactions may not be sufficient to produce protein nanocarriers with the desired stability. Variations in the environment, such as dilution or changes in pH and salt concentration, can lead to premature dissociation and/or release of the protein from the complex. Several additional interactions for improved stability can be introduced during the design of the C3Ms. Protein encapsulation with neutral-hydrophobic-charged triblock copolymers produces association colloids with higher stability. The hydrophobic block is less sensitive towards salt and pH variations and provides a barrier to protect the protein-rich coacervate [109,110,111]. Increasing the hydrophobicity of the charged block by the addition of carbon spacers between the polymer backbone and the charged side chains results in higher salt stability of protein-loaded C3Ms [109]. Association of cytochrome C with poly(ethylene glycol)-*block*-poly(aminopalmitic acid)-*block*-poly(l-aspartic acid) (PEG-*b*-PAPA-*b*-PAsp) triblock polypeptides yielded onion-like micelles with a protein-rich coacervate core, a hydrophobic PAPA shell, and a hydrophilic PEG corona [110]. The C3Ms displayed high colloidal stability towards incubation with fetal bovine serum. The cyclic RGD peptide was included in the PEG corona of the C3M to achieve specific targeting to cell-surface integrin receptors and promote endocytosis.

Chemical crosslinking of the coacervate core or (inner) shell also successfully enhances C3M stability. For example, the addition of glutaraldehyde or ethyl carbodiimide to crosslink proteins inside the coacervate stabilizes the C3Ms towards salt and pH variations [112]. However, the toxicity of these linkers lowers the biocompatibility and the irreversible crosslinking hampers the triggered release of the protein at the targeted site. This has motivated the development of reversible crosslinking strategies involving labile bonds, which can be cleaved upon specific environmental triggers, allowing the C3Ms to be stable at physiological conditions but disassemble at the site of action [113,114,115,116]. For example, crosslinking via disulphide bonds yielded highly stable C3Ms under physiological conditions. Advantageously, the cleavage of the disulphide bonds under the intracellular reducing conditions triggered the release of proteins inside the cytosol [114]. Exploiting the responsive and robust interaction between phenylboronic acid and catechol groups may also render highly stable C3Ms. Ren et al. explored this approach by preparing C3Ms using block copolymer of PEG and poly(glutamic acid), partially functionalized with phenylboronic acid (PEG-*b*-PGlu-*co*-PGluPBA), combined with a block copolymer of PEG and partially catechol-functionalized PLL (PEG-*b*-PLL-*co*-PLLCA), and cytochrome C. The micelles disassembled upon the addition of sugars as well as under acidic pH values [115]. Interestingly, both anionic and cationic proteins can be encapsulated herein, since the micelles comprise both neutral-anionic and neutral-cationic diblock copolymers. Aiming not to perturb the native enzyme and to retain its activity without chemical modifications, Chapman and co-workers instead crosslinked the polymeric shell around the enzymes [117]. First, glucose oxidase (GOx; net charge of −8) was complexed with a cationic-neutral block copolymer to form C3Ms. They showed that the shorter cationic blocks were more efficient at encapsulating GOx, and suggested that the distribution of negative charges over the protein surface may be the reason. Since the interaction that holds these C3Ms together is relatively weak, especially in solutions of elevated ionic strength (e.g., PBS), the polymer shell was crosslinked to enhance C3M stability. Chain extension of the RAFT agent using (bis)acrylamides was applied to generate a hydrogel around the enzyme. The activity of the GOx encapsulated via this non-invasive method led to high activity retention (>95%).

Crosslinking is also convenient for other biotechnological applications of enzymes, such as sensing and catalysis. Encapsulation of enzymes in crosslinked C3Ms enables the recovery and long-term, multiple usages of these biohybrid systems [118,119]. For example, C3Ms formed with a statistically copolymerized benzophenone methacrylate (BP) block copolymer (P(OEGMA-*r*-BP)-*b*-P4VP) and genetically engineered anionic alkaline phosphatase were deposited on a substrate and subsequently crosslinked under UV-light through the BP photo-crosslinkers to render an insoluble film [119]. The thin-film biosensor was utilized to detect Zn^2+^ accurately and could be stored at ambient conditions and reused multiple times. The strategy is potentially suitable to immobilize a broad spectrum of enzymes on a range of substrates. 

**Figure 5:**
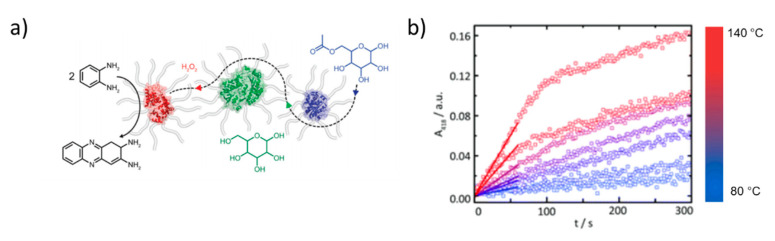
(**a**) Single enzyme nanoparticles from HRP (red), GOx (green), and lipase (blue) used for enzymatic cascades in the solvent-free phase. (**b**) Conversion of o-phenylenediamine to 2,3-diaminophenazine by HRP during the cascade reaction at temperatures > 80 °C in the absence of solvent. Adapted from Atkins et al. (doi:10.1039/c9nr06045f) as published by the Royal Society of Chemistry [128].

The impact of the local environment surrounding the encapsulated enzyme on its functionality has gained increasing attention recently. C3Ms generally contain multiple enzymes within a single core. Encapsulation into coacervates increases the local protein concentration and may promote protein oligomerization and boost activity [120]. Trypsin-loaded C3Ms exhibited up to 15 times higher enzymatic activity compared to the free enzyme. This enhancement was attributed to the partial neutralization of the imidazolium ion of the histidine residue in the catalytic triad [121,122]. Lysosome-based C3Ms also showed enhanced enzymatic activity compared to the free enzyme towards small substrates. The neutral shell of, e.g., PEG offers colloidal stability and protection against protease digestion, but it can also block access of bulky substrates to the active site. If this challenge is encountered, enzymes can be reactivated upon release from the micelles. This can be achieved in various ways, for example, by raising the salt concentration to values above the critical ionic strength, so that the C3Ms disassemble [97,123]. Alternatively, the thickness and density of the encapsulating matrix can be reduced, for example, by surface-tethering (short) polymers or growing thin, polymer shells around single enzymes to generate single enzyme nanoparticles (SENs), rather than trapping enzymes statistically into larger aggregates [117]. SENs were produced from lipase [124], GOx [125], horseradish peroxidase (HRP) [118], myoglobin [124], and ferritin [126]. Here, EDC-mediated addition of 3-(dimethylamino)-1-propylamine to the aspartic and glutamic acids generates positively charged enzymes. Electrostatically driven complexation of these supercharged proteins with alkyl-glycolic acid ethoxylated surfactants produced single enzyme-core/surfactant shell biohybrids. These discrete, single enzyme nanoparticles display a core-shell structure and are readily soluble in water and organic solvents. Freeze-drying followed by thermal annealing produced a protein-rich molten state with remarkable properties, such as exceptionally high thermal stability and solvent-free activity at very low hydration levels [127]. In these biohybrids, the PEG compartment provides a sufficiently water-like environment, while the alkyl chain increases the separation between the protein allowing for liquefaction. The high thermal stability has been exploited to perform enzymatic reactions at exceptionally high temperatures. For example, cascade reactions between HRP, GOx, and lipase were executed at temperatures up to 140 °C, in the absence of solvent (Figure 5) [128]. Interestingly, this multicomponent system was inactive below the melting transition of the biohybrids (ca. 80 °C) and increased in activity upon increasing temperature. This behavior was attributed to the temperature-dependence of the conformational ‘flexibility’ of the HRP biohybrid, the melt viscosity, and substrate diffusion.

### 3.3. C3Ms as Nano-Compartments for Small Ionic Therapeutics and Theragnostic 

Small therapeutic drugs are generally hydrophobic, nonspecific, and quickly depleted from the body due to their limited size. The therapeutic effect, therefore, relies on regular dosing, which may lead to undesirable side effects and toxicity. This has motivated the development of novel carrier systems aiming to encapsulate and deliver small molecular drugs to specific target sites, whilst minimizing adverse side effects. C3Ms have been utilized to load a variety of water-soluble, inorganic, and molecular therapeutics bearing multiple charged groups, charged drugs [129,130,131,132,133,134,135,136,137], metal complexes [138,139,140], and photosensitizers [141,142,143,144]. An overview of small therapeutics encapsulated in C3Ms is given in Table 1C. Much of the appeal of C3Ms as nanocarriers lies in their programmable nature, which enables triggered release of cargo in response to specific stimuli [17,145]. In addition, the neutral corona block endows stealth character, which increases circulation times and reduces cytotoxicity, while the rather small micellar dimensions prevent fast renal clearance. The accumulation of C3Ms within tissues is often attributed to the enhanced permeability and retention (EPR) effect. Recent studies demonstrated, however, that this EPR effect is unfortunately far less prevalent and heterogeneous in humans than in animal models (rodents) [146,147,148]. C3Ms may also be directed to specific organs by tethering suitable ligands to the corona, such as antibodies [149], folate [150], and peptides [151]. By far the most challenging criterion for the rational design and successful development of effective nanocarriers is the delicate balance between sufficient extracellular colloidal stability on the one hand, and triggered release at the desired target site on the other hand. Harnessing the intrinsic response of C3Ms to environmental changes, such as variations in salt concentrations, pH, glutathione concentration, and locally heated tumor environment, has received widespread attention as a possible means of accomplishing this central objective. 

Many strategies for triggered release, for example, of cancer drugs at tumor sites, rely on the increased acidy of the local environment of the (tumor) cells, to which electrostatically assembled nanocarriers are responsive. The anticancer drugs doxorubicin (Dox), dioxadet, and mitoxantrone can complex with neutral-anionic block copolymers to form C3Ms [132,137,152]. Interestingly, their release rate is higher at low pH due to the elevated degree of dissociation of the amine-bearing drugs. This pH-dependent release kinetics is advantageous in targeted cancer therapies, since the pH of tumor tissues is lower than that of healthy tissues. An elegant alternative approach to control the release profile of drugs is the temporal programming of C3M association and disassociation. To demonstrate the proof-of-principle, C3Ms have been exposed to controlled environments, which were induced to undergo an autonomous variation in the solution pH through the production of either base or acid, to disassemble and assemble the C3Ms within a predesignated timeframe reversibly. Specifically, C3Ms consisting of poly(*N*-methyl-2-vinylpyridinium iodide copolymer (PM2VP-*b*-PEO) and poly [9,9′-bis(3′-sodium propanoate)fluoren-2,7-yl] (cPF) were prepared at high pH in the presence of the enzyme urease, loaded with Dox, and programmed for transient disassembly by means of the urease-catalyzed hydrolysis of urea [153]. The water-soluble, conjugated polyelectrolyte cPF was negatively charged in the initial basic solution and, thus, complexed with the positively charged block copolymer. The addition of a urea-containing acidic buffer triggered the rapid dissociation of the C3Ms, as the resultant lowering of the solution pH caused protonation of cPF. Over time, the solution pH increased back to basic values as the rate of ammonia production due to the pH-dependent urease-mediated hydrolysis of urea increased. As a consequence, the micelles reassembled, and the released Dox was up retaken in the micellar core. Temporal programming of polyelectrolyte assembly can also be achieved in other ways, via, e.g., clock reactions and hydrolysis of cyclic esters, in an enzyme-based, as well as an enzyme-free, manner [154]. 

C3Ms are particularly advantageous in photodynamic therapy (PDT) and photothermal therapy (PTT) [141,142]. PDT is based on the production of reactive oxygen species (ROS) in the target tissue upon light irradiation. Conventional photosensitizers for PDT are hydrophobic aromatic molecules that self-assemble due to π-π interactions, which self-quenches the electronic excited states, resulting in reduced ROS production. To overcome the reduced efficacy of photosensitizers due to association, Kataoka and co-workers protected porphyrin photosensitizers against aggregation using a water-soluble dendritic shell and subsequently prepared C3Ms from neutral-cationic block copolymers and the anionic dendrimers with porphyrin cores [142,155,156]. Whilst the dendritic block restrained the aggregation of the porphyrins, the micellar corona also offered a protective ‘stealth’ shell to lower the cytotoxicity of the PDT. This enabled higher dosages and, thus, photochemical reactions at elevated concentrations. The mitigation of adverse side effects caused by PDT accumulation in healthy tissue was attributed to enhanced blood circulation times and EPR of the micellar formulations. 

A sparsely explored yet appealing approach to solubilize hydrophobic cargo within C3Ms is the complexation of neutral-charged block copolymers with ionic surfactants and aggregates thereof [157,158,159,160,161]. Encapsulation of surfactant micelles within C3Ms can be advantageous, since it may reduce the interactions between surfactants and bilayers, and thereby their cytotoxicity. The formulation of these multicomponent systems is a multifactorial challenge, because the interaction between the polyelectrolyte and surfactant may cause a signification reconfiguration or even disintegration of the surfactant micelle [162]. The high local surfactant concentration within the coacervate core may also induce phase transitions. External stimuli, such as salts, pH, and water-soluble molecules, can further modify the phase behavior of the surfactants [163]. Moreover, the solubilization of hydrophobic cargo can also alter the internal structure of the C3M core. Surfactant micelles may be packed in a disordered fashion within the micellar core or instead adopt liquid-crystalline ordering [16,164]. More importantly, thermodynamic considerations do not fully account for the behavior of mixtures of ionic surfactants and polyelectrolytes, as, in many cases, their association leads to kinetically trapped aggregates [165]. Disadvantageously, the solubilization capacity for the hydrophobic cargo of surfactant-polyelectrolyte C3Ms is still limited. Aiming to increase drug loading, Gradzielski and co-workers formulated macroscopic instead of microscopic coacervates containing oil/water/surfactant microemulsions [166,167]. The high oil volume fraction of microemulsions are promising for the encapsulation of large amounts of hydrophobic drugs. In the future, these macroscopic coacervates may be dispersed into stable hydrocolloids to prepare coacervate-based particles amenable to the solubilization of large amounts of both hydrophilic and hydrophobic drugs. 

## 4. Other Technological Applications

Exciting emerging applications of C3Ms also profit from the uniquely responsive nature of C3Ms and advances in controlled polymerization techniques, which enable the preparation of micelles with increasingly custom-tailored structure, stability, properties, and function. The incorporation of multivalent metal ions, spectroscopic probes, multiblock copolymers, stimuli-responsive (macro)molecules or nanoparticles, and unconventional water-soluble blocks extends the functionality of C3Ms to, e.g., diffusional probes [168,169], contrast agents and imaging probes [138,170,171,172,173], nanoreactors [174], hydrogelators [175], and crystal growth modifiers [176], and facilitates in-depth characterization at the ensemble and single-micelle level [177,178,179]. The straightforward preparation by direct dissolution from cost-effective raw materials further facilitates the translation of key concepts and innovations with high application potential into marketable technologies. A list of components used to prepare C3Ms with technological applications is given in Table 1D.

### 4.1. C3Ms as Nanoreactors and Templates

The coacervate core of the C3Ms is an ideal nanoreactor and scaffold to produce nanoparticles (NPs) with controlled size and shape. For example, multivalent metallic salts directly interact with neutral-ionic hydrophilic block copolymers to form C3Ms. Inorganic nanoparticles form upon (spontaneous) reduction or hydrolysis of the metallic salts. The C3M core limits the growth of the nanoparticles and, at the same time, provides outstanding colloidal stability. This water-based strategy to create nanoparticles is advantageous because it avoids the use of organic solvents and harmful substances. Consequently, the aqueous route is versatile, straightforward, and environmentally friendly. The addition of different chemical functionalities to the neutral block can also avoid time-consuming post-synthetic surface modifications. C3Ms have been utilized to produce various types of metallic and semiconductor nanoparticles, including metals [174,180,181,182,183,184], metal oxides [185,186,187,188,189], and quantum dots [190,191,192]. The coacervate core can also be used for biomimetic mineralization of silica [193], barium carbonate [194], and calcium carbonate [195]. Furthermore, C3Ms can template the formation of well-defined nanogels after chemical cross-linking of the core [196,197,198].

The properties of NPs produced within the C3M nanoreactors are highly dependent on the block length ratio of the ionic and neutral copolymer blocks, the overall molecular weight of the polymer, the precursor salt-to-polymer ratio, pH, and ionic strength. Interestingly, the dimensions of the NPs are not necessarily affected by the size of the micellar reactors in which the particles are produced. Instead, the action of copolymers is, in some cases, reminiscent of crystal growth modifiers. For example, the reduction of Ag ions in spherical C3Ms does not yield spherical silver nanoparticles, but elongated, silver nanowires [199]. The internal structure and composition of C3Ms may be exploited to direct the internal distribution of the produced and/or encapsulated nanoparticles [177,200]. Systematic studies on the influence of PAA molecular weight in C3Ms of PEG-*b*-PAA block copolymers complexed with HAuCl_4_ revealed that the size of the Au NPs formed after reduction was independent of the PAA block length [174,181]. In the presence of excess polyelectrolyte, the negatively charged Au NPs decorated with PEG-*b*-PAA showed outstanding colloidal stability towards salt, ionic strength, and temperature compared to common Au@citrate NPs. NP synthesis in C3Ms with block copolymers with longer PAA blocks reduced the polymer grafting density onto the NPs and concomitantly decreased colloidal stability towards physiological conditions. The subsequent addition and reduction of Ag^+^ generated Au@Ag core/shell nanoparticles with a broad surface plasmon resonance, which offered exceptionally high power conversion efficiency in solar cells [174,181].

### 4.2. C3Ms Based on Bio-Inspired Polymer Design

While the corona of the vast majority of C3Ms contains poly(ethylene oxide) chains as neutral, water-solubilizing blocks to provide colloidal stability, it may be of great interest to (partially) substitute these by other neutral, water-soluble blocks to achieve a specific function [176], impart thermal responsivity [24], or induce lateral and/or radial coronal phase segregation to generate C3Ms with a complex internal architecture [201,202]. For example, thermo-responsive blocks, such as PNIPAM, with a relatively low lower critical solution temperature, may be incorporated to tune the internal structure and colloidal stability of C3Ms [24,203]. The discovery of ice crystal growth modulation by antifreeze proteins in arctic fish inspired the development of synthetic, ice-binding polymers based on, e.g., poly(vinyl alcohol) (PVA) [204]. Interestingly, PVA-containing C3Ms can also inhibit the recrystallization of ice crystals, due to the polymer’s ice-binding properties [176]. These C3Ms may be loaded with freeze-sensitive molecules, such as proteins, to boost their stability towards freeze-thaw cycling. The coacervate core of many (but not all) C3Ms is well-known to spread on and attach to a variety of solids, including glass, metals, and oxides [205,206]. Such surfaces are, thus, readily covered with a waterborne coating upon exposure to a C3M solution [207,208]. Catechol-bearing polymers, present in mussel glue, can be used instead of PEG to favor strong adhesion to solid substrates. Robust anchoring to stainless steel surfaces was reported for micelles consisting of poly(3,4-dihydroxy-L-phenylalanine methacrylamide)-*block*-poly(dimethyl aminoethyl methacrylate) (P(mDOPA)-*co*-P(DMAEMA)) and poly(styrene sulfonate) (PSS) [209]. The addition of AgNO_3_ to the micellar solution leads to a coating formulation with high antimicrobial activity because the catechol groups reduce the silver ions to Ag^0^ and AgCl nanoparticles (DMAEMA monomers are neutralized with chloride ions) (Table 1).

## 5. Conclusions and Outlook

C3Ms hold great promise for work towards the encapsulation and preservation of water-soluble, ionic (macro)molecules, and other charged species. Their inherent responsiveness and tuneability are fundamentally exciting and attractive for applications of C3Ms in a range of technologies, including nanomedicine, coating technology, and (metal)organic nanoparticle synthesis. To fully exploit the potential of this appealing class of smart materials, it is imperative to further advance our understanding of the structure-function relations that govern their (dis)assembly pathways, (exchange) dynamics, morphology, stability, physicochemical properties, and functionalities, so that these may be fine-tuned as required for the selected application area. As the theoretical and experimental knowledge on C3Ms steadily grows, many new questions, both fundamental and applied, emerge. Advances in controlled polymerization strategies have accelerated the insight into the structure-function relations of these systems, as these greatly facilitated systematic studies of the impact of block length ratios and molecular weight on the structure, stability, properties, and functional role of C3Ms. These, and other studies on the influence of, e.g., the nature and composition of the constituent building blocks, have established a solid experimental foundation towards the development of predictive models for micellar dimensions, stability, morphology, and their variations in response to environmental cues. The elucidation of the relations between (block co)polymer architecture and C3M properties further support rational design efforts towards C3Ms with custom-tailored features and novel functionalities. Finally, we anticipate that the application of state-of-the-art characterization tools, including high-resolution imaging approaches like nanoscopy, will shed unprecedented mechanistic light in the near future on, e.g., the complex processes and interaction pathways involved in the delivery and (intra)cellular release of biologics and other drugs from C3M-based nanocarriers.

## Figures and Tables

**Table 1:** (**A**) C3Ms with Polynucleotides. (**B**) C3Ms with Proteins. (**C**) C3Ms with (Small Molecular) Drugs and Complexes. (**D**) C3Ms with Miscellaneous Oppositely Charged Species. (Morphologies: S—sphere, E—ellipsoid, C—cylinder, V—vesicle, ND—not determined).

**(A)**				
**Block Copolymer**	**Oppositely Charged Species**	**Application**	**Size (Morphology)**	**[REF]**
PEG-*b*-pAsp(DET)-Chol	pDNA	Gene delivery	120 nm (ND)	[13]
PEG-*b*-P(Lys-*co*-Lys(2IT))	pDNA	Gene delivery	100–150 nm (ND)	[14]
cRGD-PEG-*b*-p(Lys-*co*-Lys(2IT))	siRNA	Gene silencing	20 nm (S)	[37]
Lac-PEG-*b*-siRNA	PLL	Gene silencing	120 nm (S)	[38]
TGN-PEG-*b*-PDMAEMA	pDNA	Gene delivery	80 nm (S)	[39]
PEG-*b*-PEI	pDNA	Gene delivery	100–150 nm (ND)	[40]
PEG-*b*-p(Lys-*co*-Lys(FPBA))	siRNA	Gene silencing	60–80 nm (ND)	[42]
PEG-PLL	siRNA	Gene silencing	60 nm (S)	[43]
cRGD-PEG-*b*-PAsp(TEP) tetraethylenepentamine	siRNA	Gene silencing	50 nm (ND)	[45]
PEtOx-*b*-PnPrOx-*b*-PLL	pDNA	Gene delivery	100 nm (C)	[47]
PEG-*b*-(DMAEMA-*co*-BMA)	siRNA	Gene silencing	30 nm (S)	[48]
PEG-*b*-PLL	pDNA	Gene delivery	70–300 nm (C & S)	[52]
PEG-*b*-PAsp(DET) + PNIPAM-*b*-PAsp(DET)	pDNA	Gene delivery	70–90 nm (C)	[53]
Poly(galactaramidopentaethylenetetramine)	pDNA	Gene delivery	ND	[57]
Polyester-based glycopolycation	pDNA	Gene delivery	50–70 nm (ND)	[58]
poly(glycoamidoguanidine)s	pDNA	Gene delivery	60–200 nn (ND)	[59]
Poly(glycoamidoamine)s	pDNA	Gene delivery	ND	[61,85]
PEG-*b*-PEI	pDNA	Gene delivery	150 nm (ND)	[66]
PEG-*b*-Arg-*b*-PCL (polycaprolactone)	siRNA	Gene silencing	100 nm (V)	[70]
PEG-*b*-PLL	pDNA	Gene delivery	100–600 nm (C)	[73]
PEG-PLL	ssDNA & dsDNA	Fundamental	10–20 nm (S & C)	[75]
PEG-*b*-PAPTAC ((3-Acrylamidopropyl)trimethylammonium)	siRNA	Fundamental	(S, C & L)	[77]
PEG-*b*-PLL	pDNA	Gene delivery	200–350 nm	[78]
PEG-*b*-PAsp(DET)	pDNA	Gene delivery	80–600 nm (T, C)	[80]
maPEG-*b*-PLL (ma is multiarm)	pDNA	Gene delivery	200–900 nm (C)	[82]
PEG-*b*-PDMAEMA, PEG-*b*-PDMAEMA-*b*-PnBMA & PDMAEMA-*b*-PnBMA	pDNA	Gene delivery	150–100 nm (UD)	[87]
**(B)**				
**Block Copolymer**	**Oppositely Charged Species**	**Application**	**Size (Morphology)**	**[REF]**
PEG-*b*-PLL	Insulin	Protein delivery	60–200 nm (ND)	[15]
PEG-*b*-PMVP	Cyclodextrin-ferrocene host-guest	Fundamental	60 nm (S)	[17]
PEG-*b*-pAsp	PAsp(DET) + β-gal	Protein delivery	100 nm (V)	[31]
PNIPAM-*b*-PDMAEA	mCherry	Nanostructured film	20–50 nm (ND)	[88]
PEG-*b*-P2MVP	Fluorescent proteins (SBFP2, mTurquoise2, mEGFP, SYFP2, mKO2, TagRFP, mCherry)	Fluorescent probes	ca. 60 nm (ND)	[91]
PAAm-*b*-PAA	PDMAEMA + Lysozyme	Fundamental	60–80 nm (E & S)	[95]
PEG-*b*-P2MVP	PAA + Lipase	Fundamental	ca. 50 nm (S)	[96]
PEG-*b*-P2MVP	PAA + Lipase	Fundamental	40 nm (S)	[97]
POEGMA-*b*-qP4VP	PAA + Organophosphate Hydrolase	Enzymatic reactions in organic solvents	50–90 nm (S)	[99]
PEG-*b*-pAsp(DET)	Supercharged IgG	Monoclonal antibody delivery	ca. 100 nm (ND)	[101]
PEG-*b*-pAsp(DET)	Supercharged IgG + PAsp (DET)	Monoclonal antibody delivery	100–200 nm (ND)	[102]
POEGMA-*b*-PAA, POEGMA-*b*-PCEA, POEGMA-*b*-PAAVA, POEGMA-*b*-PAAOA	Lysozyme	Protein delivery	30–100 nm (ND)	[109]
PEG-*b*-PAPA-*b*-PAsp	Cytocrome C	Protein delivery	90 nm (V)	[110]
PEG-*b*-PDMAEMA-*b*-PnBMA	Cas9 protein	Gene editing	60–80 nm (ND)	[111]
PEG-*b*-PEI and PEG-*b*-PLL	Superoxide Dismutase and Catalase	Protein delivery in the central nervous system	70–170 nm (S)	[112]
PEG-*b*-PEI	Catalase	Protein delivery in the central nervous system	200 nm (ND)	[113]
PEG-*b*-PLL	Ovalbumin and Catalase + DNA	Vaccine delivery	130 nm (S)	[114]
PEG-*b*-P(Glu-*co*-GluPBA) + PEG-*b*-P(Lys-*co*-LysCA)	Insulin and Cytochrome C	Protein delivery	80–120 nm (S)	[115]
PEG-PLL and	Myoglobin and supercharged Myoglobin	Protein delivery	40 nm (ND)	[116]
PEG-*b*-(DMAPA-*co*-TreA)	Glucose Oxidase and Horseradish Peroxidase	Fundamental	ca. 10 nm (S)	[117]
Oxidized Brij	Supercharged Β-glucosidase, Supercharged Glucose Oxidase, Supercharged Horseradish Peroxidase	Enzymatic self-standing films	ND	[118]
(POEGMA-r-BP)-*b*-qP4VP	Alkaline Phosphatase	Enzymatic film	ca. 100 nm (ND)	[119]
PEG-*b*-P2VP	EGFP	Fundamental	60 nm	[120]
PEG-*b*-pAsp	Trypsin	Fundamental	70–100 nm (ND)	[121]
PEG-*b*-PAA, PEG-*b*-PGA, PEG-*b*-PMA	Trypsin	Fundamental	ND	[122]
PEG-*b*-pAsp	Lysozyme	Fundamental	55 nm (ND)	[123]
Oxidized Brij	Supercharged Myoglobin	Fundamental	ND	[124]
Oxidized Brij	Supercharged Glucose Oxidase	Fundamental	ND	[125]
4-nonylphenyl-3-sulfopropyl ether	Supercharged Ferritin	Fundamental	ND	[126]
Oxidized Brij	Supercharged Horseradish Peroxidase, Supercharged Glucose oxidase, Supercharged Lipase	Fundamental	ca. 4 nm	[128]
**(C)**				
**Block Copolymer**	**Oppositely Charged Species**	**Application**	**Size (Morphology)**	**[REF]**
PEG-*b*-P2MVP	Metallic complexes	Fundamental	60 nm (ND)	[18]
PEG-*b*-PLL	DTS + Doxorubicin	Drug delivery	150 nm (S)	[130]
PEG-*b*-PAA	Doxorubicin; mitoxantrone	Drug delivery	200 nm (S)	[132]
POEGMA-*b*-PqDMAEMA	Heparin	Drug delivery	<50 nm (S)	[134]
PEO-*b*-PDMAEMA	alkyl phosphobetaines	Drug delivery	ND	[135]
PEO-*b*-PMA	dibucaine, tetracaine, and procaine	Anaesthetic delivery	100 nm (S)	[136]
PEO-*b*-PAA	Doxorubicin; mitoxantrone	Drug delivery	200 nm (S)	[137]
PEO-*b*-PAA	Gentamicin and magnetite	Drug loading and contrast agent	80 nm (S)	[139]
POEGMA-*co*-PBAPMA	Manganese complex + Doxorubicin	Contrast agent or drug delivery	50–100 nm (S)	[140]
PEG-*b*-PLGA	ICG-PEI complex	Photothermal therapy	150 nm (S)	[141]
PEG-*b*-PLL	porphyrin dendrimer	Photodynamic therapy	40–120 nm (S)	[142]
PEG-*b*-bPEI	Chondroitin sulfate	Photodynamic therapy	150 nm (S)	[143]
PEG-*b*-PLL	Phthalocyanine dendrimer	Photodynamic therapy	50 nm (S)	[144]
PEG-*b*-PMA	Ca^2+^, crosslink, remove calcium	Monoclonal antibody carrier	150 nm (S)	[145]
PEG-*b*-PGlu	oxaliplatin (platinum drugs)	Drug delivery	30 nm (S)	[149]
PEG-*b*-PMA	Dioxadet	Drug delivery	120 nm (S)	[152]
PEG-*b*-PM2VP	Carboxylated polyfluorene-Doxorubicin complex	Drug delivery	150 nm (E)	[153]
PEG-*b*-PLys	Zn-porphyrin dendrimer	Photodynamic therapy	50 nm (S)	[156]
PEG-*b*-PMA	Doxorubicin	Drug delivery	ND	[159]
PEG-*b*-PMA	Dye or isotope labelled PAH	Diffusional nanoprobe	10–20 nm (S)	[168,169]
PEG-*b*-PM2VP	Manganese dipicolinic acid complexes	Contrast agent	25 nm (S)	[170]
**(D)**				
**Block Copolymer**	**Oppositely Charged Species**	**Application**	**Size (Morphology)**	**[REF]**
PEG-*b*-PM2VP	Lanthanide complexes	Contrast agent	20 nm (S)	[171]
PEG-*b*-PM2VP-*b*-PS	Europium complexes	Ion-specific sensor	90 nm (S)	[172]
PEG-*b*-PDMAEMA	Eu polyoxometalates	Labelling and imaging	80 nm (S)	[173]
PEG-*b*-PAA	Au precursor	Templated nanoparticle formation	100 nm (S)	[174]
PVA-*b*-PAA	PM4VP	Ice growth inhibitor	100 nm (S)	[176]
PEG-*b*-PM2VP	cPF-alt-PBT	Mechanochromic sensor	60 nm (S)	[179]
PEG-*b*-PAA	Au precursor	Gold-silver core-shell nanoparticle formation	50–200 nm (S)	[181]
PEG-*b*-PVP	PtCl_4_^2-^	Mesoporous Pt particle formation	25 nm (S)	[182]
PEG-*b*-PAA	Au precursor	Catalysis	10 nm (S)	[183]
PAAm-*b*-PAA	Mg^2+^ and Al^3+^	Formation of stable layered metal oxides	20 nm (S)	[185]
PAAm-*b*-PAA	Cu^2+^ and Al^3+^	Formation of stable layered metal oxides	50 nm (S)	[186]
PEG-*b*-PAA	Fe_2_O_3_	Porous nanocarrier, e.g., for drug molecules	200 nm (S)	[187]
PEG-*b*-PMA	(NH_4_)_2_Ce(NO_3_)_6_	CeO2 production for catalysis	ND	[188]
PEG-*b*-PAA	Ru^3+^	Formation of RuO2 as supercapacitor	ND	[189]
PAAm-*b*-PAA	Zn^2+^	Production of ZnS for optoelectronics	ND	[190]
PEG-PSCI	Cd^2+^	Production of stable CdS quantum dots	ND	[192]
PEG-*b*-PAMPS	Chitosan	Nanogel templating and formation	50 nm (S)	[197]
PEG-*b*-PAPTAC or PNIPAM-*b*-PAPTAC	Hyaluronic acid or alginate	Nanogel templating and formation	50–300 nm (S)	[198]
PEG-*b*-PAETB	PEG-*b*-PCETB	Reduction of protein adsorption on C3M-coated substrate	30 nm (S)	[205]
PPEGMA-*b*-PAA	PM2VP	Reduction of protein adsorption on silica and PS	200 nm (multi-micelle aggregates)	[207]
PmDOPA-PDMAEMA	PSS	Antimicrobial film formation by reduction of silver ions in the complexes	90 nm (S)	[209]

## Abbreviations

**Acronym**	**Full Name**	**Structure**	**Properties**
**Chitosan**	poly(d-glucosamine)		Biobased polysaccharide
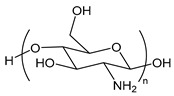
**Chol**	Cholesterol		Most abundant sterol in animals
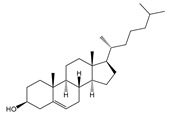
**cPF**	poly[9,9′-bis(3′-sodium propanoate)fluoren-2,7-yl]		Conjugated, solvency-dependent fluorescence emission
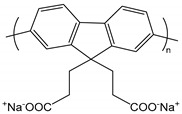
**DTS**	2,2′-dithiodisuccinic acid		Oligoanionic species; disulfide bond cleaved under reductive conditions
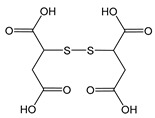
**2IT**	2-iminothiolane		Thiolating agent, reactive towards primary amines, such as in lysine
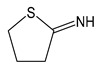
**PAA**	poly(acrylic acid)		Weak polyanion
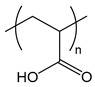
**PAAm**	poly(acrylamide)		Water-soluble, providing steric stabilization
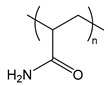
**PAAOA**	poly(acryloylaminooctanoic acid)		Charged at basic pH; amphiphilic when protonated at acidic pH
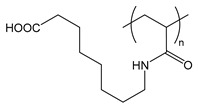
**PAAVA**	poly(acryloylaminovaleric acid)		Charged at basic pH; amphiphilic when protonated at acidic pH
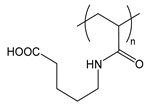
**PAMPS**	poly(2-acrylamido-2-methylpropane sulfonic acid)		Strong polyanion, can form gels
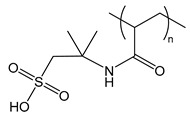
**PAPA**	poly(aminopalmitic acid)		Lipid-like, hydrophobic side chain
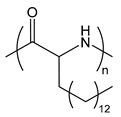
**PAPTAC**	poly(3-acrylamidopropyl trimethylammonium chloride)		Strong polycation
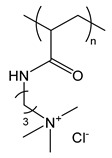
**PAsp**	poly(l-aspartic acid)		Weak anionic polypeptide
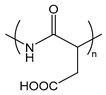
**PAsp(DET)**	poly[*N*-{*N*-(2-aminoethyl)-2-aminoethyl} aspartamide]		Cationized polypeptide, destabilization of the endosomal membrane
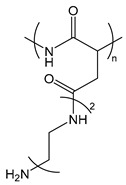
**PBP**	poly(benzophenone methacrylate)		Photo-cross-linkable sidechain
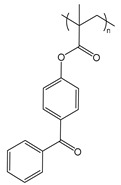
**PCEA**	poly(carboxyethyl acrylate)		Weak polyelectrolyte
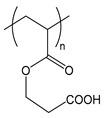
**PCL**	poly(caprolactone)		Biocompatible and relatively slowly degraded in the body
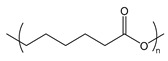
**PDMAEMA**	poly(dimethylaminoethyl methacrylate)		Strong polycation
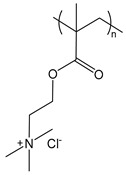
**PEG or PEO**	poly(ethylene glycol) or poly(ethylene oxide)		Biocompatible, providing steric stabilization
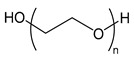
**PEI**	poly(ethylene imine)		Polycation, can also be branched; high transfection efficiency but also high toxicity
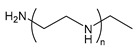
**P[F]BA**	4-carboxy[-3-fluoro]phenylboronic acid		Reversibly cross-linkable functional group
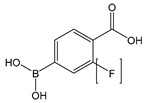
**PGA**	poly(glycolic acid)		Biodegradable polymer used for example for sutures
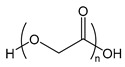
**PGAA**	poly(glycoamidoamine)		Reduced immune response and cytotoxicity and enhanced transfection efficiency; sugars and length of oligoamine can be varied (example shown)
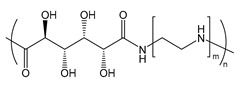
**PGlu**	poly(glutamic acid)		Anionic polypeptide
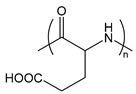
**PGluPBA**	poly(glutamicamidophenylboronic acid)		Forms reversible, covalent complexes; Lewis acid
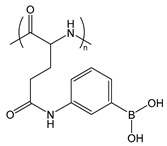
**PHis**	poly(histidine)		Provides efficient endosomal escape
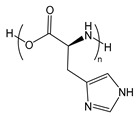
**PLL or PLys**	poly(l-lysine)		Cationic polypeptide
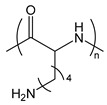
**PLLCA**	poly(ε-3,4-dihydroxyphenylcarboxyl-l-lysine)		Catechol group forms boronate ester with boronic acid under mild conditions
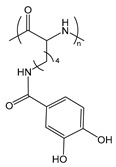
**PMA**	poly(methacrylic acid)		Weak polyanion
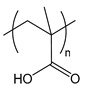
**PM2VP**	poly(*N*-methyl-2-vinylpyridinium iodide)		Strong polycation, degree of quaternization can yield varying charge densities
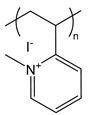
**PM4VP**	poly(*N*-methyl-4-vinylpyridinium iodide)		Strong polycation, degree of quaternization can yield varying charge densities
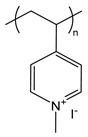
**PmDOPA**	poly(*N*-methacryloyl-3,4-dihydroxy-l-phenylalanine)		Catechol group provides adhesion to inorganic substrates (glass, metals, metal oxides)
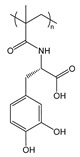
**PNIPAM**	poly(*N*-isopropyl acrylamide)		Thermoresponsive; LCST polymer forms a hydrophobic barrier at body temperature
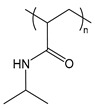
**PnPrOx**	poly(2-*n*-propyl-2-oxazoline)		Thermoresponsive; LCST polymer forms a hydrophobic barrier at body temperature
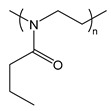
**POEGMA**	poly(oligo(ethylene glycol) methyl ether methacrylate)		Biocompatible; provides steric stabilization and anti-fouling
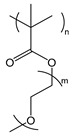
**PSS**	poly(styrene sulfonate) sodium salt		Strong polyanion
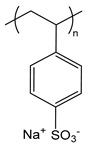
**PTreA**	poly(trehalose propylacrylamide)		Sugar moieties recognized by receptors
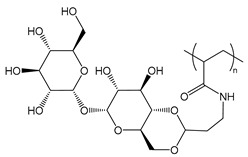
**PVA**	poly(vinyl alcohol)		Ice-binding; inhibits ice recrystallization
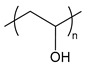
**(I)PEC**	(inter)polyelectrolyte complex		
**ATP**	adenosine triphosphate		
**BIC**	block ionomer complex		
**C3M**	complex coacervate core micelle		
**dsDNA**	double-stranded DNA		
**EDC**	1-ethyl-3-(3-dimethylaminopropyl)carbodiimide		
**EPR**	enhanced permeability and retention		
**GFP**	green fluorescent protein		
**GOx**	glucose oxidase		
**HRP**	horseradish peroxidase		
**mRNA**	messenger RNA		
**NP**	nanoparticle		
**NPC**	nuclear pore complex		
**OPH**	organophosphate hydrolase		
**pDNA**	plasmid DNA		
**PDT**	photodynamic therapy		
**PIC**	polyion complex		
**PIESA**	polymerization-induced electrostatic self-assembly		
**PTT**	photothermal therapy		
**RAFT**	reversible addition-fragmentation chain-transfer		
**siRNA**	small interfering RNA		
**ssDNA**	single-stranded DNA		
